# The development of imperfective and subjunctive marking in Hewramî

**DOI:** 10.1515/ling-2023-0247

**Published:** 2025-04-04

**Authors:** Masoud Mohammadirad, Shuan Osman Karim

**Affiliations:** University of Cambridge, Cambridge, UK

**Keywords:** analogical extension, Goranî, phonological change, verbal morphology, cyclic change

## Abstract

Contemporary dialects of Goranî (a Northwestern Iranic language spoken in Kurdistan) exhibit considerable variation in the formation of tense-aspect-mood categories. It has long been recognized that compatibility of the indicative/imperfective prefix *mi-* or the subjunctive prefix *bi-* with present-tense verb stems is phonologically conditioned. However, all attempts to identify the specific conditioning environments have failed. In this article, we reexamine the diachronic development of these affixes, showing that subsequent sound changes have obscured the original conditioning environment for the retention of these affixes. Early work on Hewramî (the main variety of Goranî) shows how the pattern of verbs that have lost the prefixes has begun to be extended to the verbs that take these prefixes for synchronically opaque reasons. The least frequent of these verbs were the first to begin the process of extension, showing inconsistent marking in these early works. Moreover, younger generations continue this process of extension using many more unmarked verbs compared to older speakers. The findings of this paper have implications for cyclic developments in the domain of tense and aspect.

## 1 Introduction

**Figure 1:**
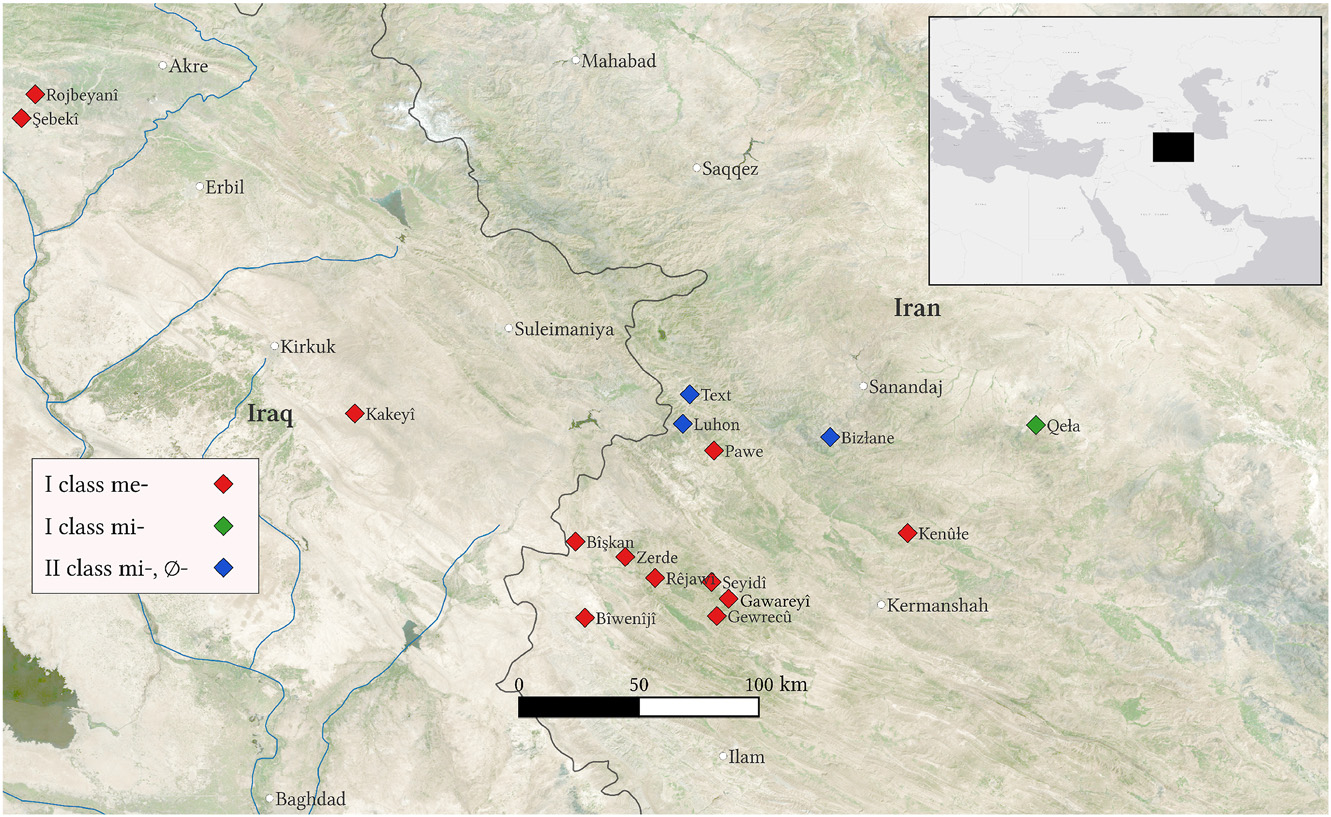
Map of imperfective markers in Goranî varieties.

The present-tense verbal conjugation in Goranî varieties is characterized by TAM prefixes, the imperfective stem, and person-number suffixes. Among the Goranî varieties, two general patterns occur in the present-tense verbal conjugation: (1) languages that feature the imperfective prefix on all verbs and (2) languages with two verb classes, verbs that take the prefix and verbs that do not. The first group can be further divided into those with the imperfective prefix *me-* and those with the prefix *mi-* featuring pretonic vowel shortening. The majority of varieties have the prefix *me-* across the board. This pattern occurs across the Goranî-speaking area. The split pattern is the second most common. However, these varieties are isolated to the Hewramî core. Finally, the varieties that take the *mi-* prefix across the board are represented by the Goranî variety of Qeła. See Figure 1 for the geographical distribution of these varieties.1Note that socio-political events have influenced the distribution of Goranî varieties, especially in the Mosul plain. The map in Figure 1 does not take recent changes into account.


Goranî is a branch of the Iranic languages (Indo-European: Iranic: Central Iranic: Northwestern Iranic: (Adharic:) Goranî). Contemporary varieties of Goranî are spoken in pockets throughout Iraqi and Iranian Kurdistan. The Goranî vernaculars are generally divided into conservative Hewramî varieties, represented in Figure 1 by varieties Text, Luhon, and Bizłane, on the one hand, and the peripheral dialects comprising the rest of the dialects in Figure 1. Note that the distinction also generally corresponds to what we have deemed above as class (2) varieties as core Hewramî, and class (1) varieties correspond to the peripheral varieties.

The languages of the Hewramî core (of Goranî), which MacKenzie (1966: 4) deemed “probably the most archaic and best preserved of the group”, retain the TAM prefixes on only a small subset of verbs. It is clear from differences in the speech patterns of speakers across generations that the shift from the class of verbs that marks TAM with prefixes to the class that marks TAM only by phonemic stress placement is ongoing; the prefix class is smaller in the language of younger speakers.

Hewramî is a member of the Goranî branch of Northwestern Iranic. It has been recognized as particularly conservative among Iranic languages, in terms of the preservation of case, number, gender, and definiteness marking on nouns, a complex system of nominal modification, and a rich system of verbal inflection.

The verbal system of Hewramî is divided into two stem types: the imperfective stem, used for the present and future tenses, as well as the imperfective aspect in the past. The perfective is employed for the past and perfect tenses as well as the conditional moods. The imperfective stem has descended from inherited verbal forms, while the past stem is the reflex of a stative participle (PIE *-to-), i.e., a nominal/adjectival form. As such, the imperfective stem features nominative-accusative alignment, while the past stem features ergative-absolutive alignment.

In Hewramî, there are two verb classes: (1) the present indicative is built by adding the prefix *mi-* to the imperfective stem and stress falls on the person-number affix, e.g., *mi-l-ó* ‘s/he goes’.2The prefix is the reflex of *ham ‘same’ and was originally one of several ways to express the progressive aspect which later became a generalized imperfective marker (following Deo 2015). (2) The present indicative consists of the imperfective stem without a prefix, and only the final stress differentiated it from other TAM categories (i.e., the subjunctive), e.g., *weró* ‘s/he eats’ versus *wéro* ‘(that) s/he eats’.

This article examines the diachronic development of the two classes of verbs, focusing on the phonological, analogical, and sociolinguistic motivations for the change. It has been recognized at least since MacKenzie (1966: 32) that phonological factors condition the presence or absence of the *mi-* prefix. However, the precise conditioning environment for the prefix had eluded scholars. The fact that the *mi-* (*m-* before vowels) and *Ø-* variants appear in complementary distribution made identifying the conditioning environment impossible. For instance, in the Hewramî variety of Luhon, the stems *waz-* and *wan-* have the same structure; the latter takes the prefix, and the former does not:

(1)

a.

**Table:** 

*mi-wan-ó*
[ind-read.prs-3sg]
‘s/he reads.’

b.

**Table:** 

*Ø-waz-ó*
[ind-request.prs-3sg]
‘s/he requests.’

Here we show that the environment that conditioned the loss of the *mi-* prefix has been obscured by regular sound changes. Once the conditioning environment was no longer apparent, these verbs became candidates for analogical extension, which can be observed in different stages of change across geography and time. We show these changes based on data collected as part of the ERC-funded ALHOME project supplemented by data from Mann and Hadank (1930), Christensen (1915), Mohammadirad (2020), Mohammadirad (forthcoming), and MacKenzie (1966).3
Mann and Hadank (1930): Bajełanî; Bîwenijî; Gehwareyî; Seyidî; Rĵabî; Christensen (1915): Pawe variety (also known as Paweyane); Mohammadirad (2020): Qeła; MacKenzie (1966): Luhon; Mohammadirad (forthcoming): Text; DOBES: Rojbeyanî


## 2 Description of imperfective TAM and negation in Goranî

In Section 1, we presented the geographic distribution of Goranî varieties, divided according to their imperfective/indicative markers. There is one class that always has an imperfective marker and one where only some verbs have an imperfective marker. Below is a brief description of these systems.

### 2.1 Class I *me-*

The most common system employs the prefix *me-* on all verbs. The varieties we have examined here that have this system are Paweyane, Şebekî, Rojbeyanî Hewlêr, Bîşkan, Bîwenîjî, Gehwareî, Kakeî, Seyyidî, Rêjawî, Gewrecû, Zerde,4Mahmoudveysi and Bailey (2013: 40) report the imperfective prefixes in Zerde to be *ma-* and *mi-*. However, they are uncertain of the phonetic realization of vowel in *me-* versus *mi-*. Our recordings from Zerde suggest that the imperfective prefix is realized as *me-*, hence Zerde belongs to Class I *me-*. and Kendûłe. The main contrast marked in the present-tense conjugation is modal, as is the case with the vast majority of Iranic languages. Verbs in the indicative mood feature the prefix *me-*, while verbs in the subjunctive mood and imperative feature the prefix *b-*.5The prefix *b-* has three allomorphs in the variety of Pawe: *b-, p-,* and *bí-*. The first occurs before single voiced consonants, the second before single voiceless consonants, and the final before consonant clusters where the second member is not a glide, e.g., *bzáno* ‘s/he knows’; *pşóro* ‘s/he washes’; and *bíjnaso* ‘s/he knows’. When the indicative is negated, the negative prefix *n-* attaches directly to the imperfective prefix *me-*, forming *ní-me-*. Note that these varieties have a complicated stress accent system. Verbal stress is characterized by a spike in amplitude and a rising f0. In the case of the stress-attracting prefix *ni-*, the amplitude spikes on the prefix but the high tone target falls on the following syllable. This reduction of Ci syllables, even when stressed, is a well-known feature of these languages (see MacKenzie 1966: 9). When the subjunctive is negated, the negation marker *né-* is attached directly to the verbal stem without the subjunctive marker *b-*. The prohibitive is marked with the negation marker *mé* without the imperative prefix *b-*. Compare the forms in Table 1, which show the forms of the indicative, subjunctive, and imperative in both the affirmative and the negative from Paweyane used as an exemplar of Class I (*me-*). The following examples partly illustrate the linguistic contexts in which imperfective and subjunctive prefixes are used.

**Table 1:** Paweyane: *kerdey* ‘do’ 2sg: ind, sbj, imp, prh.

	ind	sbj	imp/prh
aff	*me-ker-î´*	*b* ^*i*^ *-kér-î*	*b* ^*i*^ *-kér-e*
neg	*ní-me-ker-î*	*né-ker-î*	*mé-ker-e*

(2)

a.

**Table:** 

*min*	*hałac*	*dit-eke=ş*	*me-ker-im*
1sg	treatment	daughter-def=3sg:POS	ipfv-do.prs-1sg:A
‘I will cure her daughter.’			
(Mahmoudveysi et al. 2012: 101)			(Gewrecû, Goranî)

b.

**Table:** 

*dî*	*ne-tanis=şan*	*zindigî*	*bi-ker-in*
any_more	neg-can.pst=3pl:A	life	sbjv-do.prs-3pl
‘They could not live (together) after all.’			
(Mahmoudveysi et al. 2012: 168)			(Gewrecû, Goranî)

### 2.2 Class I *mi-*

The least common system employs the prefix *mi-* on all verbs. The variety we have examined here which has this system is Qela. The contrasts are the same as with the previous group. Verbs in the indicative mood feature the prefix *mi-*, while verbs in the subjunctive mood and imperative feature the prefix *bi-*. When the indicative is negated, the negative prefix *n-* attaches directly to the imperfective prefix *mi-*, causing a resyllabification *ním-*. When the subjunctive is negated, the negation marker *né-* is attached directly to the verbal stem without the subjunctive marker *b-*. The prohibitive is marked with the negation marker *mé* without the imperative prefix *b-*. Compare the forms in Table 2, which shows the forms of the indicative, subjunctive, and imperative in both the affirmative and the negative from Qela used as an exemplar of Class I (*mi-*).

**Table 2:** Qeła: *kerdey* ‘do’ 2sg: ind, sbj, imp, prh.

	ind	sbj	imp/prh
aff	*m* ^*i*^ *-ker-î´*	*b* ^*i*^ *-kér-î*	*b* ^*i*^ *-kér-e*
neg	*ní-m-ker-î*	*né-ker-î*	*mé-ker-e*

### 2.3 Class II *mi-*/*Ø-*

At the core of this study of the Goranî verbal system are the varieties with two classes of verbs: ones that take the imperfective/indicative prefix *m-* and those that do not. This group contains the varieties Bizłane, Text, and Luhon. Although these are fewer varieties than the Class I *me-* group, it should be understood that the terms Luhon and Text correspond to a large area with many villages and towns. In these varieties, the main distinction is modal just as in the previous groups. For the vast majority of verbs in Class II none of these prefixes are allowed. The indicative form is distinguished from the subjunctive only by stress placement on the final syllable, e.g., *keró* ‘s/he makes’. The subjunctive is formed with stress on the initial syllable, e.g., *kéro* ‘(that) s/he makes’. These forms are negated with the negative indicative *mé-* and the subjunctive *né-*, both stress attracting, e.g., *mékero* ‘s/he doesn’t do’ and *nékero* ‘(that) s/he doesn’t do’.

There is a smaller class of verbs that take the *m-* imperfective prefix and the *b-* subjunctive prefix. These prefixes precede all verbs that begin with vowels: e.g., *m-azó* ‘s/he lets’ and *b-ázo* ‘(that) s/he lets’. Just like Class I varieties, the indicative is negated by adding the prefix *ni-* to the verb with the indicative prefix *ni-mázo* ‘s/he doesn’t let’, and the subjunctive is negated by adding *né-* to the bare stem with vowel hiatus reduction, e.g., *n-ázo* ‘(that) s/he doesn’t let’.

The prefixes also occur before a subset of verbs that begin with consonants, e.g., *miðó* ‘s/he gives’ and *bí-ðo* ‘(that) s/he gives’. The negative of the indicative does not occur with the *n-* prefix but has the same stress attraction pattern witnessed in Class I varieties, e.g., *méðo* ‘s/he doesn’t give’. Note that this makes the negation prefix identical to the prohibitive in Class I verbs. The negative of the subjunctive is formed normally, e.g., *néðo* ‘(that) s/he doesn’t give’. The pairs in (3) illustrate the alternation in marking the imperfective, and the ones in (4) illustrate the alternation in marking the subjunctive mood, depending on the verb class. The examples are from the Text variety (Mohammadirad forthcoming).

(3)

a.

**Table:** 

***mi-l-a***	*řa=hur*
ind-go.prs-3pl:S	road.f.dir=post
‘They continued [along] the road.’	
(Mohammadirad forthcoming: JH.14)	(Hewramî, Text)

b.

**Table:** 

*m-aç-o*	*dey*	*min*	*şû-î=ş*
ind-say.prs-3sg:A	disc.ptcl	1sg	husband.m-obl.m=3sg:R
***ker-û***	*pene*		
do.prs.ind-1sg:A	to		
‘She said, “I will marry him”.’			
(Mohammadirad forthcoming: JH.59)		(Hewramî, Text)

(4)

a.

**Table:** 

*me-taw-o*	***bi-l-o***	*aweyanî*
neg.ind-can.prs-3sg:A	sbjv-go.prs-3sg:S	habitat.m
‘He wasn’t allowed to go to the village.’		
(Mohammadirad forthcoming: DG.54)		(Hewramî, Text)

b.

**Table:** 

*meger*	***ber-î***	***kîyan-î*** *=ş*	*pey*
if_only	take.prs.sbjv-2sg:A	send.prs.sbjv-2sg:A=3sg:O	to
*hewraman-î*			
pn-obl.m			
‘Maybe you could take her to Hawraman.’			
(Mohammadirad forthcoming: ZP.34)			(Hewramî, Text)

Additionally, there are verbs beginning with high or mid vowels which behave like vowel-initial verbs in the affirmative and like consonant-initial verbs in the negative. These forms are summarized in Table 3, using the forms in Luhon as an exemplar for Class II. Note that the verbs that take both the negative and indicative prefixes in the indicative have extended that form to the prohibitive.6The original form of the prohibitive was *m-ár-e* parsed as [proh-bring.prs-2sg.imp]. In analogy to the negation of present indicative, the form became *ni-m-ár-e* parsed as [neg-proh-bring.prs-2sg.imp] with double negation.


**Table 3:** Luhon: *kerdey* ‘do’ 2sg: ind, sbj, imp, prh.

		ind	sbj	imp/prh	
1	aff	*ker-î´*	*kér-î*	*kér-e*	*kerdey* ‘do’
	neg	*mé-ker-î*	*né-ker-î*	*mé-ker-e*	
2	aff	*mi-ðé-y*	*bí-ðe-y*	*bí-ð-e*	*day* ‘give’
	neg	*mé-ðe-y* ^a^	*né-ðé-y*	*mé-ð-e*	
3	aff	*m-ar-î´*	*b-ár-î*	*b-ár-e*	*awirdey* ‘bring’
	neg	*ni-m-ár-î*	*n-ár-î*	*ni-m-ár-e*	
4	aff	*m-us-î´*	*b-ús-î*	*b-ús-e*	*witey* ‘sleep’
	neg	*mé-ws-î*	*né-ws-î*	*mé-ws-e*	

^a^In post-vocalic position, the high vowels *î* and *û* become the glides *y* and *w*, respectively.

Note that there are transitional varieties belonging to this group which still retain the *ni-* prefix on verbs of the second type in Table 3, e.g., Bizłane: *nimésano* ‘he buys’ (the ALHOME recordings, Cambridge University).

#### 2.3.1 Further split

There is variation within Class II varieties in the distribution of the *m-* prefix. The class of verbs with the *m-* prefix is larger in Luhon than in the Text variety. In Table 4, we show a sample of verbs conjugated in 1sg that are in the prefix class in Luhon and the affixless class in Text:

**Table 4:** Variation in the use of *m-* in varieties with Class II verbs.

Infinitive	Gloss	Luhon	Text
*jîway*	‘live’	*mi-jîw-û´*	*jîw-û´*
*wardey*	‘eat’	*mi-wer-û´*	*wer-û´*
*nîştey*	‘sit’	*mi-nîş-û´*	*nîş-û´*
*eseřyey*	‘wipe’	*mi-seř-û´*	*seř-û*
*namyay*	‘bend’	*mi-namyé-w*	*namyé-w*
*zanay*	‘know (sth)’	*mi-zan-û´*	*zan-û´*
*řemay*	‘run’	*mi-řem-û´*	*řem-û´*
*taway*	‘be able’	*mi-taw-û´*	*taw-û´*
*wanay*, *wenay*	‘read’	*mi-wan-û´*	*wen-û´*
*yaray*	‘dare’	*mi-yar-û´*	*yar-û´*
*yaway*	‘arrive’	*mi-yaw-û´*	*yaw-û´*

Some of these examples represent subsequent phonological developments in Text, and some are analogical. We discuss the analogical forms in Section 4 and the phonological changes briefly in Section 3.1. Among the varieties we have sampled, Text has undergone the most phonological changes; the variety of Luhon represents a subset of those changes. Fewer changes have taken place in Bizłane and fewer still as one journeys outward away from the Hewramî core. We are not able in this venue to describe the changes of all Goranî varieties and thus limit ourselves to the specifics of Luhon and, where they differ, Text.

## 3 The development of the Goranî imperfective

The existence of the *m-* form prefix has long been known to Iranic linguistics because of the imperfective marker in New Persian *mı¯-*. According to Windfuhr (2009: 25–26), the Persian form originates from Old Iranic *hama-aiwa- ‘same duration, time’. In early New Persian, it began to be used to mark events in progress and continuing states. Eventually, it was generalized to the entire imperfective domain. Note that this is a process so common that its occurrence is unremarkable, known as the progressive to imperfective cycle following Deo (2015). The eventual use of the emergent progressive form as a generalized imperfective is semantically motivated and cannot be taken as proof of shared innovations. This process has occurred in Kurdish, Turkish, English, and many other languages. It occurred in the Middle Iranic Sogdian language where the combination of *ham* ‘same’ and the verbal stem preceded by the syllabic augment *a-* preserved both prefixes as *ma-* (see Windfuhr 2009: 26 and Yoshida 2009: 296). This is also the case for the Khwarezmian “pre-vocalic imperfective marker *m(a)-* < *ham + augment *a-” (Windfuhr 2009: 26). Note that in Sogdian the form Windfuhr (2009) writes as *m(a)-* is not an imperfective marker but rather a past tense formative. In Goranî, the augment only exists as part of the prefix *me-* due to boundary reanalysis. It carries no meaning separate from *m-*.7We do not mean to suggest Sogdian used *ham or the augment as an imperfective marker. Rather the same material that was recruited for this purpose in Goranî went through the same phonological changes in Sogdian, despite different functions.


Based on the quality of the vowel following *m-* in Goranî varieties, *i* or *e*, one can confidently conclude that *m-* in Hewramî is the reflex of *ham (probably with the augment *a- as in Sogdian) not *ham-aiwa as in Persian. Note that this is not a semantic shift from a past-tense formative to an imperfective marker. Rather, there is a reanalysis of the phonetic boundary, making the augment part of the imperfective marker *ham. As the original past-tense forms were all replaced by participial constructions, the augment fully demorphologized, only surviving as phonetic material on *me-*.

### 3.1 Phonological developments

As the split in Class II varieties appears to be phonologically conditioned, we must assume that all verbs originally took the *m-* prefix, and certain environments conditioned the loss of the prefix. Here, we examine the developments in Luhon and Text.

The fact that the imperfective/indicative prefix *m-* in Luhon is dependent on phonological factors was recognized by MacKenzie (1966: 32) who claimed that “[t]he factors determining which verbs do and which do not take this prefix are not evident”. He points out several generalizations. For instance, verbs that begin with *n*, *z*, *ž*, and *y* appear to take the *m-* prefix; verbs beginning with *d-*, *g-*, *ř*, and *w* appear to take the prefix. Additionally, he states that syllable structure does not seemingly play a role, citing the examples *mi-řem-*/*Ø-řež-* and *mi-wan-*/*Ø-waz-*.

Here we revisit MacKenzie’s (1966) assertions confirming that the placement of the *m-* prefix is based on phonological factors. We add to MacKenzie’s account by proposing that some of the environments conditioning the retention of the prefix, i.e., syllable structure and initial consonant, were obscured by subsequent changes.

**Analytical Notes:** In Hewramî and other regional languages, the vowel generally employed to break up illegal consonant clusters is *i* (IPA [ɪ]) or *u* (IPA [ʊ]) depending on the quality of adjacent consonants. Therefore, it is not clear whether or not instances of *i* and *u* should be understood as *Ø*. We have represented what is traditionally written as the vowel *î* as <y> when there is an adjacent vowel. That is, we consider it as a consonant in our analysis. This analysis is based on the assumption that the production of a phonologically conditioned placement of the *m-* prefix began with an unconditioned placement, and the prefix was subsequently lost in certain positions due to regular phonological processes.

The first sound change that is relevant here is pretonic reduction, where the vowel *e* is reduced to *i* before a stressed syllable. This is a widespread regional feature affecting languages like Southern Kurdish and Laki as well as core Hewramî varieties. The vowel *i* is reduced to *Ø* in most contexts except where blocked by syllable structure. The second relevant change is in forms where the *i* is lost, where the initial mC cluster is reduced to C.8Note that the creation and deletion of these clusters is difficult to track outside of the verbal system. Words featuring the necessary conditioning environment, meCVCV´ do not occur in native vocabulary. The exception to this rule is the case of an unstressed prefix *me-, only found in the verbal system. The results of these changes in Luhon are presented in Table 5 and in Text in Table 6.

**Table 5:** First-person singular imperfective/indicative Luhon.

CCV(C)					
*mi-ncen-û*	‘I mince’	*mi-jnew-û*	‘I hear’	*mi-jlêqn-û*	‘I sqaush’
*mi-řfan-û*	‘I abduct’	*mi-nwîs-û*	‘I write’	*mi-jlêwye-w*	‘I move’
*mi-jnas-û*	‘I know’	*mi-ðye-w*	‘I a given’	*mi-zye-w*	‘I arrive’
*weş-im (mi-)sy-o*	‘I like’	*mi-nye-w*	‘I put’	*Ø-kyan-û*	‘I send’
*Ø-wyer-û*	‘I pass’				

**C(V)**					

***Ø-b-û***	‘I am’	*mi-l-û*	‘I go’	*mi-z-û*	‘I give birth’
*mi-ge-w*	‘I copulate’	*mi-ðe-w*	‘I give’	*mi-ş-û*	‘I go’

**V(C)(C)**					

*m-az-û*	‘I let’	*m-êş-û*	‘I hurt’ (it.)	*m-e-w*	‘I come’
*m-aç-û*	‘I say’	*m-êşn-û*	‘I hurt’ (tr.)	*m-ûs-û*	‘I sleep’
*m-ar-û*	‘I bring’	*m-êz-w-ɔ*	‘I find’	*m-ûsn-û*	‘I put to sleep’

**y, j, z, nVC(C)**					

*mi-jîw-û*	‘I live’	*mi-yař-û*	‘I dare’	*mi-namye-w*	‘I bend’
*(mi-)jen-û*	‘I play’	*mi-yaw-û*	‘I arrive’	*mi-nîş-û*	‘I sit’
*mi-zan-û*	‘I know’				

**other CVC(C)**					

*Ø-xiłafn-û*	‘I distract’	*Ø-qêrn-û*	‘I shout’	*Ø-lałye-w*	‘I beg’
*Ø-xiw-û*	‘I laugh’	*Ø-qoz-û*	‘I cough’	*Ø-lerz-û*	‘I tremble’
*Ø-xiz-û*	‘I slide’	*Ø-fermaw-û*	‘I say’	*Ø-lês-û*	‘I lick’
*Ø-kuş-û*	‘I kill’	*Ø-fař-û*	‘I change’	*Ø-lûs-û*	‘I drink up’
*Ø-kêş-û*	‘I pull’	*Ø-fewtin-û*	‘I kill’	***mi-taw-û***	‘I can’
*Ø-kûw-û*	‘I beat’	*Ø-piř-û*	‘I fly’	*Ø-tekn-û*	‘I shake’
*Ø-ker-û*	‘I do’	*Ø-pêç-û*	‘I wrap’	*Ø-teqn-û*	‘I explode’
*Ø-kêł-û*	‘I plough’	*Ø-pêk-û*	‘I hit a mark’	*Ø-taş-û*	‘I shave’
*Ø-ken-û*	‘I uproot’	*Ø-paç-û*	‘I cut’	*Ø-ters-û*	‘I fear’
*Ø-şêl-û*	‘I press’	*Ø-pîm-û*	‘I measure’	*Ø-soç-û*	‘I burn’
*Ø-şan-û*	‘I scatter’	*Ø-pers-û*	‘I ask’	***(mi-)san-û***	‘I buy’
*Ø-şar-û*	‘I hide’	*Ø-poş-û*	‘I wear’	***mi-seř-û***	‘I wipe’
*Ø-şoqn-û*	‘I shake’	*Ø-çin-û*	‘I pick’	*Ø-wêç-û*	‘I sift’
*Ø-şoř-û*	‘I wash’	*Ø-çiř-û*	‘I call’	*Ø-wej-û*	‘I doff’
*Ø-şileqn-û*	‘I churn’	*Ø-řej-û*	‘I apply kohl’	*Ø-waz-û*	‘I request’
*Ø-biř-û*	‘I cut’	*Ø-řazn-û-we*	‘I adorn’	***mi-wan-û***	‘I read’
*Ø-bîn-û*	‘I tie’	***mi-řem-û***	‘I run’	***(mi-)wer-û***	‘I eat’
*Ø-bês-û*	‘I close’	*Ø-řês-û*	‘I spin’	*Ø-wiraz-û*	‘I sew’
*Ø-ber-û*	‘I take’	*Ø-ger-û*	‘I take’	*Ø-wureş-û*	‘I sell’
*Ø-bexş-û*	‘I forgive’	*Ø-girs-û*	‘I coagulate’	*Ø-wiz-û*	‘I throw’
*Ø-birêj-û*	‘I roast’	*Ø-girîn-û*	‘I boil’	*Ø-wîn-û*	‘I see’
*Ø-cim-û*	‘I move’	*Ø-gez-û*	‘I bite’	*Ø-mař-û*	‘I break’
*Ø-diz-û*	‘I steal’	*Ø-girew-û*	‘I cry’	*Ø-men-û*	‘I remain’
*Ø-don-û*	‘I talk to’	*Ø-gin-û*	‘I fall’	*Ø-mał-û*	‘I sweep’
*Ø-dû-û*	‘I talk’	*Ø-gef-û*	‘I bark’	*Ø-miðr-û*	‘I stop’
*Ø-dař-û*	‘I irrigate’	*Ø-gełn-û*	‘I narrate’	*Ø-mas-û*	‘I swell’
*Ø-diř-û*	‘I tear’	*Ø-mir-û*	‘I die’	*Ø-mij-û*	‘I suck’

**Table 6:** First-person singular imperfective/indicative Text.

C(V)					
*mi-ş-î*	‘you go’	*mi-l-î*	‘you go’	*mi-z-o*	‘she gives birth’
***Ø-b-û***	‘I be’	*mi-ðe-w*	‘I give’	*mi-ge-w*	‘I copulate’

**V(C)(C)**					

*m-e-w*	‘I come’	*m-az-û*	‘I let’	*m-êş-û*	‘I hurt (intr.)’
*m-êj-û*	‘I am valued’	*m-êjye-w-re*	‘I lie down’	*m-êşn-û*	‘I hurt (tr.)’
*m-ûs-û*	‘I sleep’	*m-aç-û*	‘I say’		

**CC(V)(C)**					

*mi-zn-û*	‘I create’	*mi-ðye-w*	‘I look’	*mi-nye-w*	‘I put’
*mi-zye-w*	‘I arrive’	*mi-nvîs-û*	‘I write’	*mi-jnew-û*	I hear’
*mi-jnas-û*	‘I know’	*Ø-jmar-û* ^a^	‘I count’	*Ø-vreş-û*	‘I sell’

**CVC(C)**					

*mi-san-û*	‘I buy’	Ø-jîw-û	‘I live’	Ø-yař-ű	‘I dare’
Ø-namye-w	‘I bend’	Ø-zan-û	‘I know’	*Ø-qoz-û*	‘I cough’
*Ø-lerz-û*	‘I shake’	*Ø-ger-û*	‘I grab’	*Ø-fermaw-û*	‘I say’
*Ø-pseř-û*	‘I wipe’	*Ø-kuş-û*	‘I kill, beat’	*Ø-bûş-û*	‘I kill’
*Ø-pêç-û*	‘I wrap’	*Ø-taw-û*	‘I can’	*Ø-şan-û*	‘I scatter’
*Ø-wîn-û*	‘I see’	*Ø-wêç-û*	‘I sift’	*Ø-mař-û*	‘I break’

^a^Note that the form *mi-jmar-o* occurs in the speech of older speakers; see Section 4 for more on ongoing changes.

The prefix is preserved before vowel initial roots, where coalescence prevents the separation of the prefix *m-* and the root vowel, e.g., *m-ar-ó* [ipfv-bring.prs-3sg] ‘s/he brings’. The prefix is preserved before stems that consist of a single consonant C- or a consonant and a vowel CV-, e.g., *mi-ş-û´* [ipfv-go.prs-1sg] ‘I go’ or *mi-ðé-w* [ipfv-give.prs-1sg] ‘I give’. These three categories preserved the *mi-* prefix in both Hewramî Luhon and Text with the exception of the stem *b-* ‘be/become’. The absence of the *mi-* with this verb is most likely analogical: *b-* may have lost its prefix in analogy with either the rest of *b-*initial stems, which do not take it or with the copula *hen*, which does not take it.

The prefix is retained in Luhon and Text before consonant clusters (CCV-), e.g., *mi-jnew-ó* [ipfv-hear.prs-3sg] ‘s/he hears’, where *jn* is not a possible initial cluster. There are two examples of CCVC verbs that do not take the prefix *mi-*: *wyer-ó* [ipfv-pass.prs-3sg] ‘s/he passes’ and *kyan-ó* [ipfv-send.prs-3sg] ‘s/he sends’. These two apparent exceptions to the rule are likely conditioned by syllabification. Essentially, the glide can be the second member of a complex onset *kyV*, as two-stop consonants cannot. The verb *mi-ncen-ó* [ipfv-mince.prs-3sg] ‘s/he minces’ would syllabify *min.ce.no*,9Note that MacKenzie glosses the word as ‘chop’ in Luhon. There is no difference in usage between Luhon and Text despite the difference in form. Here, ‘chop’ and ‘mince’ are synonyms’. whereas a hypothetical *mikyano would syllabify *mi.kya.no.10Note that the analysis *mi.kya.no is based on the fact that Cy sequences behave like C sequences phonologically. Therefore, the Cy-initial verbs *mi-sye-*, *mi-ðye-*, *mi-nye-*, and *mi-zye* behave like C(V) verbs taking the prefix. The verbs *wyer-* and *kyan-* pattern like CVC verbs and do not take the prefix.11Note that this type of syllabification is cross-linguistically common, e.g., in Greek, Latin, Japanese, etc., although it is not ubiquitous. For instance, in Japanese, Cy clusters are tolerated, e.g., *gyūniku* ‘beef’, but all other syllable initial clusters are impossible *gdu, *gru, *gwu, etc. Likewise, Moder Greek syllabifies glides as part of the onset consonant, e.g., *i.ɣya* ‘health’ (not *iɣ.ya, Deligiorgis 1988: 16). The strategy for dealing with these initial consonant clusters in Text is different from Luhon. For instance, the Luhon stem *-řfan-* receives a prosthetic *bi-* 12This is the first study to recognize the existence of the verbs in Text with a non-etymological *bi-* prefix in the indicative. This is most likely a result of the *bi-* of the subjunctive/imperative becoming reanalyzed as part of the stem. Note that this type of boundary reanalysis is common among Iranic languages, including all of Kurdish, e.g., the neighboring Southern Kurdish variety of Qurwe, which has reanalyzed the old imperfective prefix *di-* as part of *h-*initial verbs, e.g., *e-tê-m* [ipfv-come.prs.ipfv-1sg ‘I come’. in Text, adding it to the prefixless class: *biřfano* ‘s/he abducts’. The Luhon stem *-ncen-*, drops its initial segment *n* in Text, becoming part of the affixless class: *cenó* ‘s/he minces’.

The category with the most variation is stems beginning with CVC syllables, where consonant quality dictates the presence or absence of the prefix *mi-*. CVC(C) stems beginning with *y*, *j*, *z*, and *n* always occur with the prefix *mi-* in Luhon but without it in Text. The rest of CVC characters do not take the prefix in either variety. There are but a few exceptions to this rule that must be explained: Luhon: *miseřó* ‘s/he wipes’, *miwanó* ‘s/he reads’, *miweró* ‘s/he eats’, *miřemó* ‘s/he runs’, and *mitawó* ‘s/he can’, Luhon and Text: *misanó* ‘s/he buys’.

The sibilants show the *m-* prefix with several roots *miseřó* ‘s/he wipes’, *weş-misyó* ‘be pleasant’, and *misanó* ‘s/he buys’. Of these, only *misyó* has a syllable structure that conditions the prefix *mi-*, cf., *miðyó* ‘s/he sees’ and *miðó* ‘s/he gives’. However, there are etymological clues that point to an original conditioning environment that has been all but lost to time.

The forms *miseřó* ‘s/he wipes’ and *misanó* ‘s/he buys’, have the syllable structure sVC, which should not condition the presence of the *mi-* prefix, cf., *Ø-soç-ó* ‘s/he burns’. However, the past-tense stem and infinitive show an irregularity where a prosthetic vowel *e-* 13Note that MacKenzie (1966: 32) treats the prosthetic *e-* as part of the stem that is reduced to *i-* (i.e., lost) when preceded by a prefix in all present-tense forms. This assertion is not supported etymologically. It has no parallel in other Goranî varieties or other Iranic languages more generally. It does not have any parallels elsewhere in Luhon. is added to the stem, i.e., *esáy* ‘to buy’ and *eseřyéy* ‘to wipe’. This is a feature of consonant-cluster-initial roots in Luhon, e.g., nasal-stop: ***e*** *ncenyéy*/*mincenû´* ‘to mince/I mince’, trill-voiceless fricative: ***e*** *řfáy*/*miřfan’ˆu* ‘to snatch/I snatch’, voiced fricative-nasal: ***e*** *jnasáy*/*mijnasû´* ‘to know/I know’, ***e*** *jnewyéy*/*mijnewû´* ‘to hear/I hear’. This pattern implies that the forms ***e*** *seřyéy* and ***e*** *sáy* acquired their prosthetic vowels because of an original cluster. This is additionally supported etymologically. The verb *misanó* ‘s/he buys’ is from Proto-Iranic *staHn (Cheung 2006: 361), which suggests an original *st cluster subsequently reduced to *s* (likely, with an intervening *ss stage) late in Hewramî. Note that these clusters are reduced even in borrowed words, e.g., *des* ‘hand’ from Persian *dast*. Likewise, *miseřó* ‘s/he wipes’ is cognate with Northern Kurdish *strîn* ‘to be freed from’, showing the same reduction of the *st cluster: *misteřo > (*misseřo >) *miseřó*.14Note that Cheung (2006: 336) has a different etymology. He claims that ***e*** *seřyéy* is cognate with Sanskrit *chard-* (Cheung 2006: 336). This does not affect our assertion here as the initial *ch* is the reflex of PIE *skˆ clusters. PIE *kˆ becomes the affricate *ts in Proto-Iranic and is affected by preconsonantal spirantization becoming *θs and eventually becoming *θ* in Southwestern Iranic and *s* elsewhere including Hewramî (for more on this shift, see Karim 2023). That implies an original *ss cluster *msseřo, which gets the anaptyctic vowel inserted between the *m-* prefix and the root, i.e., *misseřo. Subsequently, the geminate cluster is reduced to *miseřó* preserving the *m-* prefix in the present and the prosthetic *e-* in the infinitive ***e*** *seřyéy*. We think that Cheung’s (2006) etymology is incorrect. However, our analysis here does not hinge on this detail. It was suggested by an anonymous review that another possibility is that this verb is related to Kurdish *sirran/stirrîn*, which most likely derives from *us-tarH (Cheung 2006: 382). What is clear is that there was an original consonant cluster. In Text, these verbs are sometimes integrated into the system by the same strategies used in the CCV group. For instance Luhon *miseřó* corresponds to Text *pseřó* ‘s/he wipes’. This reflects the insertion of the prosthetic *bi-* prefix with subsequent devoicing. The verb *misanó* ‘s/he buys’ remains unchanged in Text.

CVC syllables beginning with the phoneme *w* condition the loss of the *m-* prefix in Luhon and Text. However, in Luhon, the prefix occurs with certain verbs where just like *esáy* ‘to buy’, the original structure was obscured by subsequent changes. Root-initial *w* can be from Proto-Iranic *hw (*miwanó* ‘s/he reads’, Cheung 2006: 145), *xw (<*kw) (*wazó* ‘s/he requests’, Cheung 2006: 460), *w (*wyeró* ‘s/he passes’, Cheung 2006: 381), or *f (*wureşó* ‘s/he sells’, Cheung 2006: 429). The *xw -and *f-initial segments follow the same rules as *x-* and *f-*initial segments; the *m-* prefix is deleted across the board; in other words *xw and *f changed to *w before the loss of *m(i)-*. Likewise, the verbs with roots beginning in *w* form PIr. *w, lose the *m-* prefix across the board like the other bilabials *b*, *p*, and *m*.

Only the roots with an initial *w* from *hw can take the *mi-* prefix. This is treated like an initial consonant cluster. The first type takes the *mi-* prefix and loses the *h*, yielding the forms *miwanó* ‘s/he reads’ and *miweró* ‘s/he eats’. In contrast, the other two *hw examples lose the *m-* prefix. The verb *wurnó* ‘s/he demolishes’ derives from the Old Iranic root *hwar (cf. Avestan *xwara*, Cheung 2006: 150). This has an identical root structure to *miweró* ‘s/he eats’ (cf. Avestan *xwar-*, Cheung 2006: 148). However, there are two very different outcomes, both in terms of the presence or absence of the prefix *mi-* and in terms of the vowel quality of the stem. The sequences *ur* and *er* reflect the zero- and guna-grade of the Old Iranic root, respectively. One could make the argument that the difference in the structure of the verbs that take the prefix and those that do not is what is responsible for the difference. However, this change may not be phonological at all, as the verbs *wurnó* and *wizó* are perhaps too infrequent to support the irregular conjugation. This is essentially a single root *wer-* ‘eat’ that behaves contrary to expectation. We know, based on the regularity of sound change, that there is either a conditioned phonological explanation or an analogical one. This root has an etymon that supports both possibilities. As such, we do not push either solution to a point of only minor significance to our overall study.

The only verbs that begin with rhotics or laterals l, ł, r, and ř that take the prefix *mi-* do so because of syllable structure, e.g., CCV: *mi-řfan-ó* ‘s/he abducts’ and C:15The retention of the prefixes with single consonant roots is likely the result of stress. For instance, the verb *kerdey* distinguishes imperfective from subjunctive through stress placement, e.g., *keró* [do.prs.ipfv.3sg] and *kéro* [do.prs.sbjv.3sg]. This distinction would be neutralized with C(V)-, Cy(V)- roots, e.g., *ló.
*mi-l-o* ‘s/he goes’. The one possible exception to this rule in Luhon is the *miřemó* ‘s/he runs’. This verb taking the *mi-* prefix appears to go against the rules. However, this may also have an etymological explanation. Cheung (2006: 312) shows that *ram, the etymon of *miřemó*, shows the root variant *θram in some Iranic languages (perhaps from *ati-*ram.), suggesting an original consonant cluster from high to low sonority, i.e., *hř. In this case, *miřemo* would be in the same category as *misano* and *miseřo*.

The last of the six verbs that irregularly show the *mi-* prefix in Luhon is *mitawó* ‘s/he can’. This is the only verb beginning with a voiceless stop that takes this prefix. Its presence is quite likely to be analogical. There is a widespread regional tendency for the verbs ‘want’, ‘know’, and ‘be able’ to develop irregularly together (see Chyet 2024). These verbs take on features of each other’s irregular conjugation. For example, Northern Kurdish features the verbs *zanîn* ‘to know’ and *karîn* ‘to be able’. Each optionally can occur without the present imperfective/indicative prefix *d(i)-* becoming *zanim* ‘I know’ and *karim* 16According to Cheung (2006: 237–238), the Kurmancî verb *karîn* ‘to be able’ is the causative form of *kirin* ‘to do’. However, this assertion does not match the phonetic expectation for a causative, which might be *kerandin/kerîn -or *kandin/kîn -depending on the reduction of *r* on the imperfective stem. In addition to the phonological problems, the semantic shifts from ‘make do’ to ‘be able’ are not entirely clear. One hypothesis of the current author for this form is that *kar-* is a direct borrowing from Armenian *gar*. Western Armenian *g* being from an original *k*. Of course, contact between Armenian and Iranic languages is widely attested; see Clackson (2017), Macak (2017), etc. The *n* of the *kan* stem observed in some dialects would then be further contamination from the verb to know *zan-* (following the hypothesis of Chyet 2024). ‘I can’ (Haig and Öpengin 2018: 125). There are corresponding negative forms *nizanim* ‘I do not know’ and *nikarim* ‘I can’t’ for expected *nazanim and *nakarim. Finally, both verbs employ the form of the past subjunctive for the present subjunctive *zanibim* ‘(that) I know’ and *karibim* ‘(that) I can’ for expected *bizanim and *bikarim. These are the only verbs that behave in this irregular fashion in Northern Kurdish. Likewise in Western Armenian, the verbs *kidanal* ‘to know’ and *garenal* 17See Note 16. ‘to be able’ share an irregular conjugation. The present indicative of both occur without the typical imperfective/indicative prefix *gə-* and the *-n* present tense extension, i.e., *kidem* ‘I know’ and *grnam* ‘I can’ for expected *gəkidanam and *gəgarenam. This tendency also spans the entire range of attested language in the region as the Akkadian verbs *īde* ‘I know’ and *īše* ‘I want’ have a matching irregular conjugation; the expected forms are *ēdê and *ēšê, respectively. Although, the form of ‘I can’ in the Oldest Akkadian shows the expected *alaʔa* but in later texts, it merges with ‘to know’ and ‘to want’, i.e., *īle*.

The verb *zanáy* ‘to know’ in Luhon begins with *z* which always takes the *m-* prefix. We hypothesize that the only exception to the loss of *m-* before voiceless-stop-initial roots *tawáy* ‘to be able’ and *mitawó* ‘s/he can’ has regained the *m-* prefix in analogy with the verb *mizanó* ‘he knows’, following a larger regional trend.

An additional verb that should be discussed in the context of the CVC pattern is *kuştéy*. In Text and Luhon, it belongs to the CVC category and rather unremarkably loses its *mi-* prefix. However, in certain contexts, the stem *kuş-* has a variant where the vowel *u* is reduced to labialization on the previous segment, i.e., *k* ^*w*^ *ş-*. In the Hewramî variety of Bizłane, older speakers treat that stem as the base form belonging to the CCV type and therefore, conditioning the prefix, e.g., *mu-k* ^*w*^ *ş-û* ‘I kill’.

### 3.2 The distribution of *n-* in Hewramî

Old Iranic has two verbal negation markers *na and *mā. The former was used more generally, while the latter was used with multiple forms to express the prohibitive, i.e., the negative imperative. One striking feature of Hewramî is that the form of the prohibitive seems to have taken over the function of the negative in nearly all forms. The verbs that do not take the *m-* prefix, e.g., *keró* ‘s/he does’, feature the stressed negative prefix *mé-*, e.g., *mékero* ‘s/he doesn’t’. Consonant initial verbs that take the *m-* prefix also use the prefix *mé-* for negation, e.g., *miðó* ‘s/he gives’ and *méðo* ‘s/he doesn’t give’. It is also used with high/mid-vowel-initial verbs (all of which take the *m-* prefix), e.g., *mûsó* ‘s/he sleeps’ and *méwso* ‘s/he doesn’t sleep’.

In contrast, a small number of verbs take a reduced form of the *na negator *ni-*. All of these verbs take the *m-* prefix, e.g., *maró* ‘s/he brings’ and *nimáro* ‘s/he doesn’t bring’, *mo* ‘s/he comes’ and *nimo* ‘s/he doesn’t come’, etc. In examination of these forms, it becomes clear that the apparent similarity to the prohibitive *mé-* does not necessarily suggest that the prohibitive was extended to the negative. In fact, the prohibitive of these low-vowel-initial verbs has been borrowed from the indicative, e.g., *ni-m-ár-e* [neg-ind-bring.prs-2sg.imp].

The *n-*form negative is essentially ubiquitous; it is used exclusively in the past, with the present subjunctive, and formerly with the indicative as well (as observed in other Goranî varieties, see Section 2.1). In contrast, the *m-*form marker was restricted to the prohibitive. It seems odd that such a marginal morpheme like the prohibitive would replace an extremely common one in this way. In fact, a comparison of forms in other Goranî varieties preserves a medial stage suggesting that the prohibitive form is actually borrowed from the indicative and not the other way around. The closely related Paweyane and Bizłane provide good evidence for this proposal.

In Paweyane, the indicative prefix is *me-* except when the vowel *e* coalesces with a stem-initial vowel, e.g., *mezanó* ‘s/he knows’ and *maró* ‘s/he brings’. The negative *ni-* is added to the indicative stem attracting stress, becoming *nímezano* ‘s/he doesn’t know’. In Bizłane, the *ni-* prefix attracts stress but is not able to host it, e.g., *n* ^*i*^ *mézano* ‘s/he doesn’t know’ and *n* ^*i*^ *máro* ‘s/he doesn’t bring’. These forms are clearly different from the prohibitive *mézane* ‘do not know!’ and *máre* ‘do not bring!’ that show the prohibitive prefix *mé-*, which is different from the negative prefix *n- ´*. Note that in the cluster *nm, the same environment that conditions the loss of *m* in Luhon, is satisfied, i.e., *nmézano > *mézano* ‘s/he doesn’t know’ and *mmenó > *menó* ‘s/he remains’. The similarity between *mé-* [prh-] and *mé-* [neg.ind-] is therefore a phonological merger not extension. In the small set of low-vowel-initial verbs that would have preserved the negative prefix as *ni-* and preserving a negative indicative-prohibitive distinction (Paweyane: *nimáro* ‘s/he doesn’t bring’ and *máre* ‘do not bring!’), the indicative is extended to the prohibitive resulting in syncretism, e.g., Luhon/Text: *nimáre* ‘do not bring!’

### 3.3 The distribution of *b-* in Hewramî

The prefix *bi-* is nearly ubiquitous within New Western Iranic languages. Its appearance is unremarkable. The origin of the *b-* prefix is somewhat more complicated than that of *m-*. According to Joseph (1975), *be*, although phonologically identical to dative case marker (i.e., the preposition ‘to’), has an independent etymon, the preverb *ui ‘away’. This etymological proposal is problematic in Persian as *wi prefixes become *go-* (following Horn 1895: 64). It is equally problematic for Hewramî, where this shift did not take place. For instance, the verb *wirazo* ‘s/he sews’ is from Proto-Iranic *ui-*Hraz (Cheung 2006: 198). Note that this is the same preverb thought to be the predecessor of the subjunctive prefix *b-* following Joseph (1975). Joseph’s (1975) proposal has been revisited several times, e.g., Morgenstierne (1927); Gershevitch (1964); Skiærvø (2009); Brunner (1977); Sims-Williams (1996), see Jügel (2013: 30–33) for a full overview.

Jügel (2013) proposes a tripartite etymology with origins in *apa-, *b^h^e(ǵ^h^), and *b^h^ē/b^h^ō. The form *apa- contributes primarily to the nominal system as part of forms like *abī* ‘without, less’, *bī* ‘without, less’, and *bīrūn* ‘outside’. The forms *b^h^e(ǵ^h^) and *b^h^ē/b^h^ō contribute primarily to the verbal system as the paritcle *be-* and its phonologically conditioned New Persian allomorphs *bi-* and *bo-*. Jügel (2013) proposes that these forms influenced each other in terms of semantics and phonology.

In Hewramî, the reflex of the preverb *apa- is the ambifix *(-)ewe(-)* prefixed in the infinitive in Luhon and suffixed elsewhere in the grammar.18MacKenzie (1961: 84) relates this form to New Persian *bāz* and colloquial New Persian *vā*, both known cognates of *apa, the former with the *ič extension. Also, see Durkin-Meisterernst (2014: 227) for Middle Persian cognates *ab*, *aba*, *abe*, *af*. For the semantic shift from ‘away/backward’ to iterative, see Belelli (2022). This shows that *apa is not a proper candidate for the etymon of Hewramî *b-* as it shifted to *w* through regular sound changes. This does not clash with Jügel’s (2013) proposal, which states that *apa had a semantic influence on the verbal formatives, not necessarily phonological. One can assume *aba- as a medial stage between *apa- and *(-)ewe(-)*. The current study does not aim to confirm or deny Jügel’s (2013) proposal, which is a tangential issue. We add one point to the assertion that “further studies are necessary”. Various phonological changes in New Iranic languages that differ from Persian can help narrow the possible etyma for *b-* and many other forms. For instance, the Hewramî form *b(i)-* could not be the result of *ui or *apa, which surface as *wî* and *ewe* in Hewramî, respectively.

The subjunctive prefix bi- tends to appear with the same set of roots that take the *m-* prefix, suggesting parallel development of these TAM prefixes. The *bi-* prefix is stress-attracting but cannot host it, as is clear in Paweyane, where it always occurs. Compare Paweyane *pşóro* ‘(that) s/he washes’ and Luhon *şóro* ‘(that) s/he washes’. This is true of verbs that retain the prefix in Luhon as well, e.g., *bisáno* ‘s/he buys’. The verbs that lose the prefix go through two sound changes: (1) the prefix attracts stress to the stem vowel. Then, (2) the initial cluster is reduced, and the only trace of the prefix is the stress on the first syllable.

It is, however, notable that there is not a perfect overlap in the distribution of *bi-* and *mi-* as shown by the Text data in Table 7. By way of example, the root de- ‘give’ takes the *m-* prefix, but the subjunctive form can be either *bi-ðó* or *dó* ‘(that) s/he gives’. There is also variation in the other direction. The present stem *wer-* ‘eat’ belongs to the class of verbs that do not take the *m-* prefix. However, some speakers would use the subjunctive *bi-*, see Table 7.

**Table 7:** The distribution of *mi-* and *bi-* in H. Text.

Gloss	indicative	subjunctive
‘bring’	*m-aró*	*b-áro*
‘come’	*m-ê´*	*b-ê´*
‘let’	*m-azó*	*b-ázo*
‘sleep’	*m-ûsó*	*b-û´so*
‘hurt’	*m-êşó*	*b-ê´şo*
‘go’	*mi-ló*	*bi-ló*
‘go’	*mi-şó*	*bi-şo*
‘give birth’	*mi-zó*	*bi-zó*
‘create’	*mi-znó*	*bí-zno*
‘copulate’	*mi-gó*	*bi-gó*
‘give’	*mi-ðó*	*bi-ðó/do*
‘look at’	*mi-ðyó*	*bi-ðyó*
‘leave’	*mi-zyó*	*bi-zyó*
‘write’	*mi-nvîsó*	*bí-nvîso*
‘put’	*mi-nyó*	*bi-nyó*
‘buy’	*mi-sanó*	*bi-sáno*
‘do’	*Ø-keró*	*Ø-kéro*
‘be able’	*Ø-tawó*	*Ø-táwo*
‘spare’	*Ø-sparó*	*bí-sparo*
‘run’	*Ø-řemó*	*(bi)-rém-o*
‘eat’	*Ø-weró*	*(bi)-wér-o*
‘say’	*m-açó*	*Ø-wáço*

## 4 Analogical change and shift toward regularization among younger generations

The factors that created the complex verbal class system in varieties like Hewramî Luhon, Text, and Bizłane are mostly phonological. However, there have been several analogical developments (see Section 3). There are now subsequent examples of analogy, where verbs that originally belonged to one class now belong to another. The tendency for verbs to jump classes was apparent in variation already at the time of MacKenzie (1966). Here we describe this variation among speakers in Luhon as shown in MacKenzie (1966), among older speakers in Text and Bizłane, and differences as can be observed between older and younger speakers in data collected in Text and Bizłane.

When MacKenzie (1966) interviewed his consultant in 1957, there were already verbs that showed variation as to whether or not they took the *mi-* prefix. Uncertainty as to whether or not the verbs *(mi-)wer-û* ‘I eat’ and *(mi-)san-û* ‘I buy’ should take the prefix *mi-* is understandable. CVC verbs beginning with *w* or *s* do not typically take the prefix. The original conditioning environments for these two verbs were CCV clusters *stan- and *hwer-, respectively. Native speakers, unaware of the historical layer, would see these verbs as exceptions to an internalized rule. Both *weş-im (mi-)sy-o* ‘I like’ and *(mi-)jen-û* ‘I play’ belong to classes CyV and jVC, which condition the presence of the prefix. However, *weş-im (mi-)sy-o* ‘I like’ is the only verb that begins with *s* belonging to this class, and the vast majority of voiceless fricative-initial verbs do not take the prefix. Likewise, *jenû* may be joining the majority of CVC verbs as the prefixless class. It is worth noting that the only one of these verbs to occur with the prefix in Text is *misano* ‘s/he buys’.

There is some cross-dialectal evidence of verbs changing classes. In Luhon, the verb *řemay* ‘run’ takes both modal prefixes: *mi-řem-o* ‘s/he runs’ and *bi-řemo* ‘(that) s/he runs’. In the speech of the older generation in Text, the *mi-* prefix is lost but the subjunctive *bi-* is retained: *Ø-řemo* versus *bi-řemo*. This shows that in Text, the verb ‘run’ has partially joined the affixless class triggered by analogy with other *ř*-initial verbs, e.g., *Ø-řêsû* ‘I spin’; *Ø-řêş-û* ‘I put on kohl’. The verb ‘to eat’ shows the opposing developments in Luhon and Text. In Luhon, the *bi-* prefix is lost in the subjunctive, but the *m-* prefix is optionally used in the indicative, *wéro* ‘(that) s/he eat’ versus *(mi-)wer-o* ‘s/he eats’. In Text, the *m-* prefix is lost in the indicative, hence *weró* ‘s/he eats’, and the *bi-* prefix is retained optionally in the speech of the older generation, *(bi)-wer-o* ‘(that) s/he eat’. In both varieties, the verb ‘to eat’ is on its way to joining the affixless class. However, the path taken is different.

There is further evidence that verbs shifting classes is an ongoing change. The Hewramî Text data in Table 8 come from Mohammadirad’s fieldnotes as part of his Hewramî grammar (Mohammadirad forthcoming). The Bizłane data are from the recordings collected as part of the ALHOME project. As can be seen, the young generation tends to extend many more verbs to the affixless class. This is especially true of verbs that take the *m-* prefix for synchronically opaque reasons. This can be seen for indicative and subjunctive verbs in Table 8. The direction of change is from the less frequent affixed class to the more frequent affixless class.

**Table 8:** Generational differences in the class of verbs.

Variety	Example	Oldgeneration	Young generation
Bizłane	‘I know’	*(mi)-şnas-û*	*Ø-şnas-û*
Bizłane	‘I give’	*(mi)-ðe-w*	*Ø-de-w*
Text	‘Run!’	*bi-řeme*	*Ø-řeme*
Text	‘Eat!’	*(bi-)wer-e*	*Ø-were*
Text	‘Spare!’	*(bi-)spar-o*	*Ø-sparo*

## 5 Regional parallels

The development of the *m-* and *b-* prefixes has parallels in the languages of the region. Matras (2010) takes the development of the imperfective prefix in Persian, Kurdish, Armenian, Neo-Aramaic, and Levantine Arabic as one piece of strong evidence in favor of an East Anatolia linguistic area. Haig (2014: 19–26) gives a critical overview of this feature, stating that the imperfective prefix is either missing in many languages in the core East Anatolia region, e.g., Zazaki, Turkish, various dialects of Neo-Aramaic, or is limited to occur with certain verbs, as in the Trans-Zab dialects of Neo-Aramaic.

Indeed, the development of Hewramî *m-* has parallels with neighboring Neo-Aramaic dialects. For instance, in the Jewish Neo-Aramaic dialect of Sanandaj, the imperfective particle *k-*, assumed to be historically derived from the presentative particle *ka, occurs almost exclusively with verbs that have ʔ and h as root-initial consonants, e.g., *ʔ-w-l* ‘to do’ versus *k-ol* ‘he does’. Khan and Mohammadirad (2024: 155–164) show that the indicative prefix *k-* in the Jewish Neo-Aramaic dialect of Sanandaj exhibits matching in its distribution with the imperfective *m-* in Hewramî.

There are additional regional parallels all over the Iranian world. In nearby Kurdish varieties, there is such a development with the most frequent *h*-initial roots. In the Northern Central Kurdish Mukrî variety present imperfective/indicative of *hatin* ‘to come’ is *dê* ‘s/he comes’ (Öpengin 2016: 46). This is transparently a combination of the prefix *de-* and the present stem *(h)ê* that loses its initial *h* (PIE H_2_ following Kümmel 2014) word-medially. However, in the Central Kurdish variety of Kerkûk, the imperfective prefix *e-* has lost its *d* through the regularization of a sandhi variant (following the analysis of Karim 2024 based on Bybee 2002, 2003). The *d* lost elsewhere has been preserved in the form *dê* where it has unimorphated with the stem. The same is true in Southern Kurdish, where even more diversity of imperfective marking exists.

In Southern Kurdish, both the verbs *hatin* ‘to come’ and *hawirdin* ‘to bring’ have present-tense stems that are unimorphated with the *de-* prefix. In the Southern Kurdish variety of Qurwe, the *de-* prefix has generalized the sandhi variant becoming *e-*; the verbs in question surface as *tê* ‘s/he comes’ and *têrê* ‘s/he brings’ (< *d-hêr-) with the original *d* devoicing before the stem-initial *h* that was lost. Another peculiarity of the Qurwe variety is that the younger generation of speakers has recently started to extend the regular *e* marker to these verbs that already have the *t* reflex of the original marker becoming *etê* ‘s/he comes’ and *etêrê* ‘s/he brings’.19These observations are based on Masoud Mohammadirad’s field notes. Note that Fattah (2000) only shows the form with the extension of the common imperfective marker *e-* to the imperfective stem with the dental variant, e.g., *etêrê*. We know of the form *têrê* in the speech pattern of older Qurwe residents because one of the current authors is a native speaker of Qurwe (SK). In the Southern Kurdish variety Sencebî, the imperfective marker has been lost in the present tense due to the extension of a sandhi form. Despite this total loss of *de*, the dental variant has been preserved on the two verbs, e.g., *tyam* ‘I come’ and *tyarim* ‘I bring’ (for all Southern Kurdish forms of come and bring, see Fattah 2000: 360). There are a few important facts about the distribution of these markers in Southern Kurdish: based on Fattah’s (2000) data, there are seven varieties of Southern Kurdish that only show the imperfective marker as part of the unimorphated negative marker *nye* (< *ni-de) as well as on the verbs ‘to come’ and ‘to bring’ (Arkwâzi, Dušayx, Iwân, Kaprât, Mandıli, Sarpol, Šerwân). There are a further eight that have the same distribution but have preserved the suffixed element of the bipartite Kurdish imperfective marker (Kałhor (Shahabad), Čamčamâł, Harasam, Kırmanšâh, Qasırıširin, Sanjabi, Xâłesa, Šerwân Kıłâwây). There are a further ten that have lost the imperfective marker in the present tense, only showing the imperfective marker as part of the unimorphated negative marker *nye* (< *ni-de) as well as on the verbs ‘to come’ and ‘to bring’, but have retained the prefix in the past tense (Xânaqin, Malıkšâhi, Myaxâs, Ilâm, Mıhrân, Rikâ (Sarna), Sâleh Âbâd, Warmızyâr, Zurbâtiya, Kordali). It can be said that the second group, which shows no prefix in the present but a suffixal form in the past, most clearly resembles the Hewramî pattern (Table 9).

**Table 9:** Šerwân Kıłâwây and Hewramî Luhon: ‘to bring’ and ‘to do’.

	ŠK	HL
‘s/he brings’	***t-*** *yeri(d)*	***m-*** *aro*
‘s/he doesn’t bring’	*nye-* ***t-*** *yeri(d)*	*ni-* ***m-*** *aro*
‘s/he used to bring’	*hawird* ***-ya***	*ar* ***-ê***
‘s/he didn’t use to bring’	*nyehawird* ***-ya***	*nar* ***-ê***
‘s/he does’	***Ø-*** *kê(d)*	***Ø-*** *kero*
‘s/he doesn’t do’	*nye-* ***Ø-*** *kê(d)*	*me-* ***Ø-*** *kero*
‘s/he used to do’	*kird* ***-ya***	*ker* ***-ê***
‘s/he didn’t use to do’	*nyekird* ***-ya***	*neker* ***-ê***

This loss of the imperfective marker, except where it unimorphates with a stem for phonological reasons, is also found in Balochi. The imperfective marker *k-* occurs only with roots beginning with *h*, and the imperfective prefix *a-* is found with all verbs (Nourzaei and Jahani 2012). This phonological conditioning points to a wider distribution that narrowed because of regular sound changes. The Baluchi verbs that commonly take the *k-*type imperfective marker are *(k)ayəg* ‘to come’, *(k)arəg* ‘to bring’, *(k)ylləg* ‘to leave, abandon, let go’, *(k)oštəg* ‘to stand’, and *(k)wškynəg* ‘to listen, hear’ (Barker and Mengal 2014: 133–134).20The full form of the original imperfective marker can be gleaned from the extant allomorphs *a-*, *k-* (before *h*), and *ar-* (before *r*, some varieties), e.g., *ar=rant* ‘they go’ (Nourzaei and Jahani 2012: 175). The marker *k-* occurs only with *h*-initial verbs similar to what is observed in Kurdish (perhaps as a reflex of the *kar imperfective marker found in the Caspian languages following Karim 2020).


This phenomenon is also found in the so-called Central Plateau Dialects. Stilo (2007) shows forms in several varieties that have regularized a marker *e-* or *a-* (similar to Kurdish *e-*), which have lost the dental element and reinstate it before what he calls “vowel-initial” stems. However, these are the same set of verbs that preserved the dental prefix in Southern Kurdish and the *k-* prefix in Balochi. Examples from Stilo (2007) showing the juxtaposition of C-initial stems and V-initial stems include Jowšaqani: *a-pic-am* ‘I twist’, *but at-ār-am* ‘I bring’; Qohr. *a-k-ūn* ‘I fall’, *but at-ār-ūn* ‘I bring’; Tāri *a-ker-õ* ‘I do’, but *at-ār-õ* ‘I bring’; Aby. *e-kar-ān* ‘I do’, but *et-özmar-ān* ‘I count’; Ards. *e-ker-õ* ‘I do’ but *et-oroš-õ* ‘I sell’. See discussion in Windfuhr (1991: 242–250); Krahnke (1976: 182–187) also comes to a similar conclusion. Just as the move southward in the Kurdish-speaking region, Stilo (2007) shows that moving eastward amongst the Central Plateau Dialects, the imperfective/indicative marker disappears in most contexts, e.g., *biri, biri, bira* ‘I, you, he takes/carries away’, or *der-k-i, der-k-i, der-k-a* ‘I, you, he falls’, but the original *-t* still shows up as a remnant before vowels (in the durative tenses): (present) *tāri, tārém (= t-ār-i, t-ār-ém)* ‘I, we bring’ or *tosi (= t-os-i)* ‘I get up’ and imperfect *ši-t-ārt* ‘he would bring’. We keep Stilo’s (2007) original parsing here, although we side with the analysis in Karim (2024) that these should be parsed as *a-t-ār-am* [ipfv-ipfv-bring.prs-1sg.A] or even more accurately as *a-tār-am* [ipfv-bring.prs.ipfv-1sg.A] with the *t* now part of a present imperfective stem. This analysis is additionally suggested by further examples that show the dental form becoming part of the present-tense stem regardless of aspect or mood, e.g., “Qohr. *tengas-/tangašt* versus Meym. *enges-/angašt* ‘look’, Soi *angis-* [pres.] …Thus while the Qohrudi present tense is *a-tangisun* ‘I look’ – almost identical to Soi *at-angisom* – the Qohrudi subjunctive, *bátengisun* (*bá-tengis-un*) and the preterit *batangaštun* (= *ba-tangašt-un*) show that the initial *t-* is now part of the root (but not in Soi)” (Stilo 2007).

In a sense, the forms of Qohrudi have followed this shift to the furthest analogical conclusion. Once regular phonological rules delete the imperfective prefix from all but a handful of verbs, the salience of the distinction decreases. This is further bolstered by the progressive-to-imperfective shift which takes a meaningful distinction in the present, progressive versus imperfective aspect, and replaces it with a redundant distinction, indicative versus subjunctive. We call this distinction redundant because the subjunctive is marked with the *b-* prefix that could contrast with an unmarked indicative form. These two forces led to the reanalysis of the imperfective prefix as simply a part of a suppletive imperfective stem on some verbs, as in Southern Kurdish, and then generalized as a present-tense stem, as in Qohrudi.

In contrast with Qohrudi, etc., Hewramî retains distinctions in several, albeit restricted, contexts, making it clear that the *m-* formatives are, in fact, affixes and not just part of a suppletive stem. However, there are a few forms in the Text variety that show that the *bi* prefix has been reanalyzed as part of the present tense stem, just like the imperfective prefix in Qohrudi and some Qurwe (SK) speakers, e.g., *biřfano* ‘s/he abducts’ and *pseřo* ‘s/he wipes’.

## 6 Discussion and conclusion

The tendency for TAM prefixes and the imperfective prefix, in particular, to be lost due to phonological change is a widespread feature among Iranic languages and their neighbors. Hewramî is no exception to this tendency. When observing the Goranî languages as a whole, it is clear that every stage of this development has been preserved:

– Pretonic vowel reduction:
  The majority of Goranî varieties show the imperfective prefix *me-*, e.g., Paweyane, Şebekî, Rojbeyanî Hewlêr, Bîşkan, Bîwenîjî, Gehwareî, Kakeî, Seyyidî, Rêjawî, Gewrecû, and Kendûłe. This prefix reduces to *mi-* in Qela and Zerdeyane, eventually becoming the only reflex of the imperfective marker. The variety of Bizłane preserves the distinction between pretonic reduction and the unreduced form, i.e., *mi-son-û´* [ipfv-buy.prs-3sg] versus *ni-mé-son-û* [neg-ipfv-buy.prs-1sg].
– Loss of *i* even in word-initial stressed syllables creating consonant clusters:
  According to MacKenzie (1966: 9), initial syllables with *i* or *u* are reduced even when stressed. An underlying form like *bisano surfaces as *b* ^*i*^ *sáno* ‘(that) s/he buys’ often with retrogressive voicing assimilation. This also occurs with this combination of negative and indicative in forms like *nimáro* ‘s/he does not bring’. In the variety of Bizłane where the intermediate stage described above is preserved, the same pattern can be observed: *ní-me-son-û > *n* ^*i*^ *-mé-son-û* [neg-ipfv-buy.prs-1sg].
– Clusters lost in some varieties:
  As described in Section 3, various phonological changes caused vowel reduction leading to illegal word-initial consonant clusters. In the varieties of Luhon and Text, this caused the loss of the imperfective, subjunctive, and negative markers in most contexts, leaving the stress-attracting properties of the subjunctive markers as the only remnant of its existence, and it left both the stress-attracting property of the negative and the original unreduced imperfective marker *mé-* as the host of the stress for the negative marker. An original set like *me-ker-ó, *ní-me-ker-o, *bí-ker-o underwent reductions, becoming *me-ker-ó*, *ní-me-ker-o*, ***p*** ^***i***^ ***-*** *kér-o* as observed in Paweyane. Subsequent pretonic reduction led to *m ^**i**^ **-**ker-ó, *n^i^-mé-ker-o, *p^i^-kér-o, the system observed in Bizłane for verbs that retained the prefixes, e.g., *m* ^*i*^ *-son-á*, *n* ^*i*^ *-mé-son-a*, *p* ^*i*^ *-són-a.* Later cluster reduction produced the system observed in Text and Luhon ***Ø-*** *ker-ó*, ***Ø-*** *mé-ker-o*, ***Ø-*** *kér-o*, where stress placement is the only remnant of the original markers.

Variation between verb class membership among the core Hewramî varieties gives us a window into ongoing developments within this group. We have been lucky enough to have access to field recordings collected as part of the ERC-funded ALHOME project, featuring multi-generational speech samples and metalinguistic analysis. It seems that younger speakers are aware that their speech is different from that of their parents’ generation. One speaker from Text commented that their father would say *biřeme* ‘run!’ where they could just say *řeme* ‘run!’

The generational differences show us that verbs switch classes gradually one cell at a time. A verb that takes both the imperfective and subjunctive prefixes like *miðew* ‘I give’ and *biðew* ‘(that) I give’ may lose the subjunctive prefix while retaining the imperfective, e.g., *miðéw* ‘I give’ and *déw* ‘(that) I give’. In contrast, a verb from the same class could lose the imperfective prefix and keep the subjunctive *sparû´* ‘I spare’ and *bísparû* ‘(that) I spare’.

Finally, the findings of this paper have implications for cyclic developments in the domain of tense and aspect. Deo (2015) suggests that the progressive to imperfective shift may be cyclic, in the sense that once a progressive marker turns into an imperfective marker, the speakers may innovate a new device for expressing progressive, which could result in another grammaticalization cycle, hence “the cycle may begin anew” (Deo 2015: 14:47). In the case of Hewramî, the progressive *ham acquired imperfective meaning early on, thus going through the progressive-imperfective cycle before it disappeared. In the current state of the language, an innovative reduplicated construction expresses the progressive, see (5). The progressive construction consists of the inflected form of the verb preceded by the full stem reduplicant and the formative *-ay*. E.g., Text: *wer-ay-wer-û* [redup-prog-eat.prs-1sg] ‘I am eating’. Note that stems that preserve the *m-* treat it as part of the stem reduplicant. *mil-ay-mil-û* [redup-prog-go.prs-1sg] ‘I’m going’.

Recent research by Mohammadirad (forthcoming) suggests that the reduplicated construction may be used to express time-bounded future events, as in (5). In addition, the construction may be used with negative and interrogative clauses.

(5)

**Table:** 

*êşew*	*gêł-ay*	*gêł-a*	*pey*	*pîya-ya*
tonight	search.prs-nmlz	search.prs.ind-3pl:A	for	man-obl.pl
‘Tonight, they will be looking for men [who can serve them].’				
(Mohammadirad forthcoming: JL.34)			(Hewramî, Text)	

This may suggest that the reduplicated construction, which is approaching the categorical progressive stage of Deo (2015), is also spreading into the imperfective domain. The expression of the future acts as a step towards finally extending to time-bounded and habitual events. The data thus shows that, for Hewramî, the progressive to imperfective cycle has begun anew. This is inline with Deo’s (2015) hypothesis of cyclicity.
